# Method for simultaneous selection of treatment isocenters and margins for polymetastatic extracranial stereotactic ablative radiotherapy

**DOI:** 10.1002/acm2.70681

**Published:** 2026-07-01

**Authors:** Jordan M. Slagowski, Gemma A. Davies, Adam Bayliss, Laura C. Bennett, Mustafa M. Basree, Michael F. Bassetti, Carri K. Glide‐Hurst

**Affiliations:** ^1^ Department of Radiation Medicine University of Wisconsin‐Madison Madison Wisconsin USA; ^2^ Department of Medical Physics University of Wisconsin‐Madison Madison Wisconsin USA

**Keywords:** extracranial SABR, isocenter optimization, margins, polymetastatic disease

## Abstract

**Background:**

Stereotactic ablative radiotherapy (SABR) represents a new era of treatment for polymetastatic extracranial disease but introduces unique challenges in isocenter selection and margin optimization to balance treatment efficiency and normal tissue sparing.

**Purpose:**

Develop an end‐to‐end method to simultaneously cluster M tumor targets and optimize isocenter number (N) and position to minimize the additional margins required to maintain target coverage as tumor‐to‐isocenter distance increases.

**Methods:**

K‐means clustering identified candidate isocenters ranging from one to one per target. Margins required for 95% coverage probability were determined based on tumor‐to‐isocenter distance, accounting for translational (5 mm) and rotational (1°, 2°, 3°) uncertainties modeled as three‐dimensional normal distributions. K‐means total margin volume was compared versus isotropic 5‐ and 10‐mm margins and benchmarked against a derivative‐free hybrid optimization method. The method was evaluated on 20 clinical lung cases with 2–21 targets per patient, target‐to‐target distances of 3.8–32.4 cm, and volumes of 0.07–41.05 cm^3^.

**Results:**

The total margin volume determined by k‐means showed no significant differences (*p* = 0.94) relative to the hybrid optimization method, with median differences of 0.03% (0.01 cm^3^), 0.14% (0.06 cm^3^), and 0.19% (0.15 cm^3^) for 1°, 2°, and 3° rotational uncertainties, respectively. Significant (*p* < 0.0167) differences in total margin were observed versus fixed margins of 5 or 10 mm. Increasing the number of isocenters for a given patient reduced the median total margin volume by 31.0% for *N* = 2 versus *N* = 1 and 15.7% for *N* = 3 versus *N* = 2, but diminishing returns were observed as additional isocenters were added (*N* = 4 to *N* = 6: −6.6%, −5.4%, and −3.7%, respectively). Median run time was 0.19 s for k‐means versus 306.9 s for the reference method.

**Conclusion:**

K‐means clustering offers an efficient method for selecting the number and locations of isocenters to reduce the total margin volume in multi‐target extracranial SABR.

## 1 INTRODUCTION

Metastatic tumors account for >80% of cancer deaths and continue to be the primary cause of cancer‐associated mortality.^1^ Improved patient outcomes with ablative treatment to all sites of metastatic disease has been demonstrated.^2^ The SABR‐COMET trial demonstrated improved overall survival for patients with up to 5 metastatic lesions treated with stereotactic ablative radiotherapy (SABR) to all tumor sites versus standard of care.^3^ The SABR‐COMET‐10 trial expanded inclusion criteria to 10 lesions.^4^ The phase 1 ARREST study demonstrated safety and feasibility to treat patients with polymetastatic disease for >10 lesions.^5^ The thrust to treat increasing numbers of tumor targets introduces unique technical and clinical challenges to facilitate efficient and accurate treatment planning strategies.

Extracranial SABR is most commonly performed with an individual treatment plan and isocenter located within each target to reduce setup uncertainty and margins, as margins must increase with target distance to isocenter to compensate for rotational uncertainties.^6^, ^7^, ^8^ However, treating each target with an individual isocenter may not be practical in patients with multiple metastatic targets, motivating techniques for single‐isocenter multi‐target (SIMT) lung treatments that may improve efficiency, enhance patient comfort, and increase the feasibility of treatment completion.^9^, ^10^, ^11^

Extending SIMT techniques from intracranial to thoracic sites introduces additional challenges due to larger target volumes, respiratory and cardiac motion, and tradeoffs between organ‐at‐risk sparing, margin size, and ablative tumor doses.^10^ Respiratory motion and repeated breath hold may not be feasible on the time scale of several hour‐long treatments due to patient discomfort and baseline drift. These challenges are compounded by the potential for larger distances between tumors in the thoracic region leading to a loss of target coverage and higher doses to organs‐at‐risk due to increased rotational errors.^6^, ^11^

A statistical model by Chang provides a method to determine the additional PTV margin required to maintain a set probability of target coverage as a function of target distance to isocenter assuming translational and rotational errors.^7^, ^8^ Several studies have proposed isocenter selection strategies in the context of intracranial disease, using stochastic and heuristic optimization techniques to maximize target coverage or minimize total margin volume for multiple brain metastases.^12^, ^13^, ^14^ Existing methods have largely focused on *intracranial* treatments and selecting the *single* isocenter that minimizes the total margin volume, whereas in the *extracranial* setting, multiple isocenters are often required.^15^ Yock et al. described a method to group multiple brain targets to one or more isocenters using the k‐means clustering algorithm,^16^ motivating us to explore the impact of isocenter location on total treatment margin for thoracic patients.

The purpose of this work was to develop and validate a strategy to select isocenter locations, cluster tumor targets, and minimize total margin volume for multi‐target thoracic SABR. The method simultaneously selects the optimal number (N) and positions of isocenters to efficiently treat multiple (M) tumors with N ≤ M, and was validated against a hybrid optimization technique. In addition, our method optimizes the minimal margin required to prevent a loss of coverage probability as target distance to isocenter increases, thereby delivering an end‐to‐end solution for isocenter and margin assessment in complex multi‐target cases.

## 2 METHODS

### 2.1 Statistical margin determination

Radiotherapy treatment plans are optimized to deliver the prescribed dose to the planning target volume (PTV), an expansion of the CTV that accounts for random and systematic uncertainties to ensure a specified probability of CTV coverage. For isocenters placed within the CTV, rotational uncertainties may be safely ignored and compensated for using image guidance. As the distance, d, of the isocenter to CTV increases, rotational errors, θ, produce translational errors which must be accounted for in single‐isocenter (or N ≤ M) multi‐target treatments.

A statistical formalism to determine the margin required to account for translational and rotational errors was described by Chang.^7^, ^8^ Translational and rotational setup errors of the CTV about the isocenter are assumed to follow independent three‐dimensional normal distributions with standard deviations $\sigma_{S}$ (mm) and $\sigma_{D}$ (degrees), respectively. Rotational uncertainties are treated as residual errors about the isocenter that are not fully corrected during initial setup, resulting in an effective revolution of the CTV about isocenter. The additional translational error induced by rotational uncertainties for a target *j*, at distance $d_{i,j}$, from isocenter i, is $\sigma_{j,R}=0.816d_{i,j}\sigma_{D}\frac{\pi }{180}$. The CTV to PTV margin is provided by Equation 1 where $\chi_{\alpha }^{2}$ is the chi‐square value for significance level α for a three degrees of freedom Chi distribution. For this work, $\chi_{\alpha =0.05}$= 2.795 which ensures the CTV has a 95% probability of receiving the prescription dose.

(1) $$m_{j}=\chi_{\alpha }\;\sqrt{{\sigma_{S}}^{2}+{\sigma_{j,R}}^{2}}=\chi_{\alpha }\;\sqrt{{\sigma_{S}}^{2}+{\left(0.816d_{i,j}\sigma_{D}\frac{\pi }{180}\right)}^{2}}$$

The value of $\sigma_{S}$, was set equal to $\frac{5\;mm}{\chi_{\alpha =0.05}}$ such that in the limit *N* = M, $d_{i,j}≅0,\;\sigma_{j,R}=0$, uniform 5 mm treatment margins are applied, as used in the SABR‐COMET trial.^3^ However, $\sigma_{S}$ is a tunable parameter that should be set based on institution specific setup, image guidance, and motion management protocols. Rotational errors were varied with $\sigma_{D}$ set equal to 1°, 2°, and 3° to reflect a clinically relevant range of setup uncertainties as used in a prior single‐isocenter SBRT study.^11^ Thus, the margin to account for rotational errors increases as a function of target distance to isocenter.

### 2.2 Optimized isocenter selection

**FIGURE 1:**
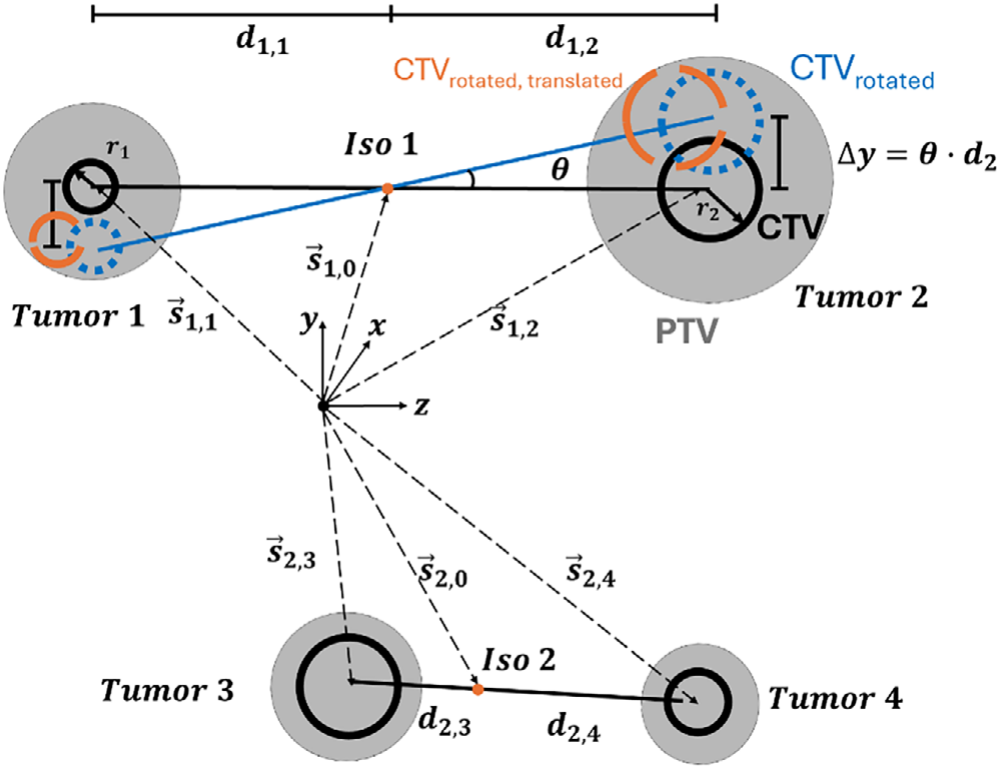
The isocenter selection problem and notation for multi‐target thoracic SABR is presented.

Figure 1 presents the multi‐target isocenter selection problem for a hypothetical patient presenting with four target volumes (M = 4) and two isocenters (*N* = 2). The location of isocenter, i, is defined by coordinates $\left(x_{i},\;y_{i},\;z_{i}\right)$ within a three‐dimensional Euclidean space. The location of isocenter, i, relative to the global coordinate system origin, *0*, is defined by vector ${\vec{s}}_{i,0}$. Similarly, the location of the target CTV, j, is defined by coordinates $\left(x_{j},\;y_{j},\;z_{j}\right)$ and vector ${\vec{s}}_{i,j}$ where i is the assigned treatment isocenter for target j. The distance, $d_{i,j}=∥{\vec{s}}_{i,j}-{\vec{s}}_{i,0}∥$, between the target, j_,_ and isocenter, i, is given by Equation 2.

(2) $$d_{i,j}=\sqrt{{\left(x_{j}-x_{i}\right)}^{2}+{\left(y_{j}-y_{i}\right)}^{2}+{\left(z_{j}-z_{i}\right)}^{2}}$$

The isotropic CTV to PTV margin expansion, $m_{j}$, in units of millimeter, may be determined for a target with an assigned isocenter using Equation 1. The volume of margin about a spherical CTV with radius, $r_{j}$ is given by Equation 3.

(3) $$V_{m_{j}}=\frac{4}{3}\;\pi \left[{\left(r_{j}+m_{j}\right)}^{3}-r_{j}^{3}\right]$$

The total margin volume, V, for a series of M lesions may be computed as the summation of the individual target margins according to Equation 4. The total margin volume is a function of the number of tumor targets (M), the number of treatment isocenters (N), the radius of each target, $r_{j}$, the position of each isocenter, ${\vec{s}}_{i,0},$ and the location of each target relative to its assigned isocenter, ${\vec{s}}_{i,j}$.

(4) $$V\;\left(r_{j},\;{\vec{s}}_{i,j},\;{\vec{s}}_{i,0},M,N\right)=\sum_{j=1}^{M}V_{m_{j}}$$

The isocenter selection process consists of identifying the N isocenter positions that minimize the total volume of margin required to compensate for translational and rotational errors given the positions of each target.

(5) $$\hat{{\vec{s}}_{i,0}}=\underset{{\vec{s}}_{i,0}}{\mathrm{argmin}}V\left(r_{j},\;{\vec{s}}_{i,j},\;{\vec{s}}_{i,0},M,N\right)$$

Isocenter positions that minimize the total treatment margin volume were determined using a hybrid optimization method to provide a reference for comparison and validation of the heuristic clustering method described in section 2.3. Initial candidate isocenter positions were randomly initialized across a set of scenarios, ranging from a single isocenter (*N* = 1) to a unique isocenter for each individual target (*N* = M). In each iteration, the M tumor targets were assigned to one of the N candidate isocenters based on the shortest target‐to‐isocenter distance. Simulated annealing with a Boltzmann temperature schedule was then applied to iteratively refine the candidate isocenter coordinates, with the goal of minimizing the total treatment margin volume. To mitigate the risk of converging to a local minimum, and to increase the probability of finding the global optimum, a total of 1,000 restart procedures were employed, each with randomized initial isocenter positions. To further reduce the probability of converging to a local minimum, isocenter locations were independently optimized using a Nelder‐Mead simplex algorithm, and a pattern search method.^17^ The final set of isocenters, yielding the lowest total margin volume, was selected for subsequent treatment planning.

### 2.3 Isocenter selection via k‐means clustering

**FIGURE 2:**
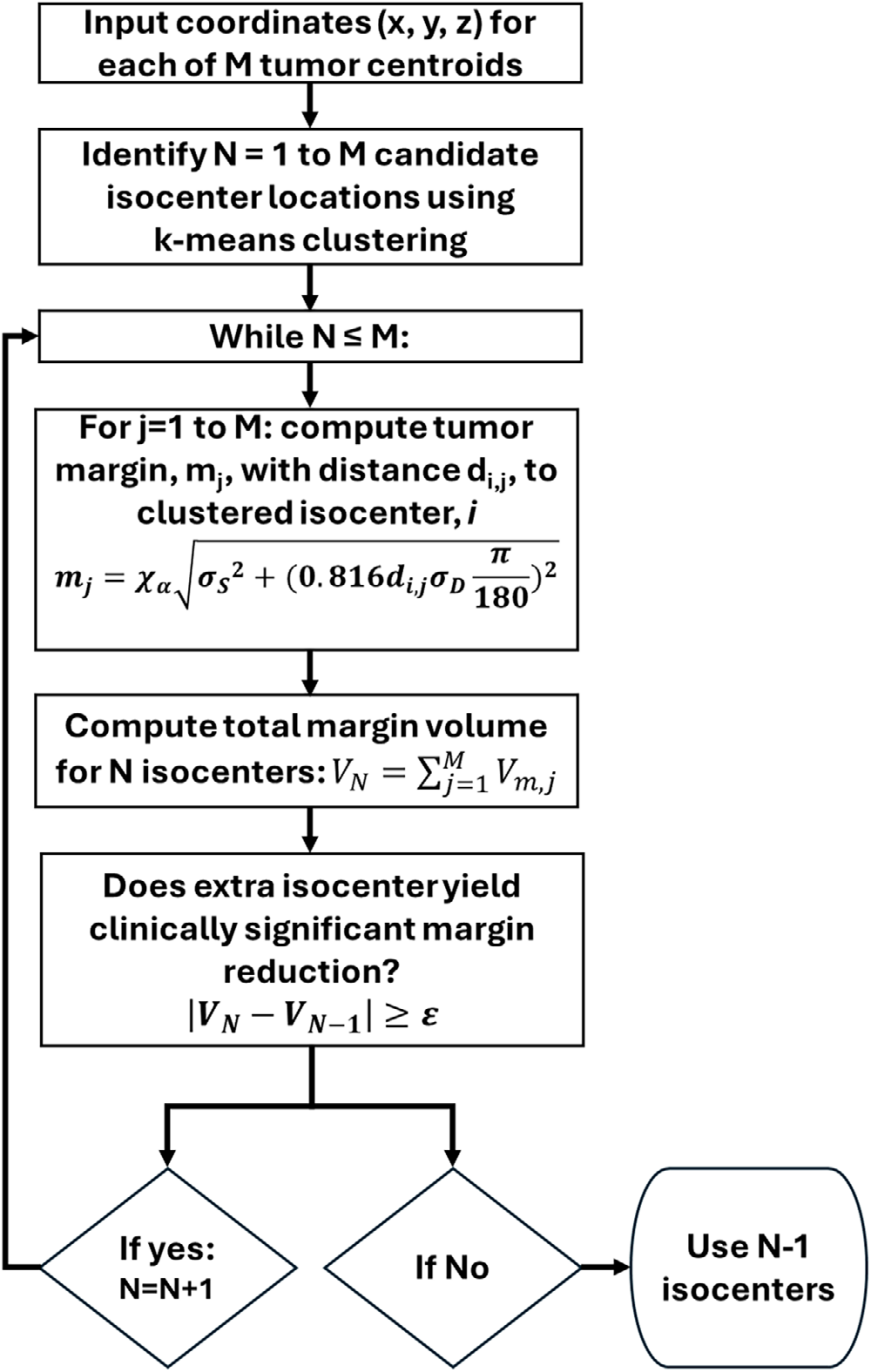
Flow chart for automatic selection of isocenters and treatment margins.

Because simulated annealing based isocenter selection becomes computationally intensive as the number of targets and isocenters increases, we explored k‐means clustering as an alternative. K‐means clustering partitions M targets into N clusters that minimize the squared Euclidean distance. Due to the total margin volume dependence on Euclidean distance to isocenter, we hypothesized that k‐means clustering could be applied to efficiently determine near optimal isocenter locations for multi‐target thoracic SABR. The automatic isocenter selection and margin determination algorithm is summarized in Figure 2. The algorithm accepts a list of centroid coordinates ($x_{j},\;y_{j},\;z_{j}$) for each of M tumor targets. A set of candidate isocenter locations ranging from *N* = 1 to *N* = M are then determined using the k‐means clustering method (implemented using *kmeans* in MATLAB R2023b) to group each of the M tumor targets. Individual target margins and total margin volumes are computed for each candidate group of isocenter positions using Equations 1 and 4, respectively. The algorithm is terminated when it is determined that an additional isocenter does not reduce the total margin volume by a clinically significant amount. This logic is implemented by selecting the set of *N‐1* treatment isocenters when $|V_{N}-V_{N-1}|<\varepsilon$. The termination parameter, ε, may be set as an absolute margin volume or as a relative percentage. In this work, the optimal number of isocenters balancing treatment efficiency and margin reduction was selected as the minimum N after which the percentage reduction in total margin for an additional isocenter was < 10%. Sensitivity to the threshold parameter was evaluated by also considering values of 20% and 30%. The exact value of ε should ultimately be determined in collaboration with a radiation oncologist or through treatment planning studies. For comparison, the percentage difference of the k‐means determined total margin was computed versus the hybrid optimization method for each patient case and set of rotational errors.

### 2.4 Patient characteristics

Twenty patients with multiple thoracic tumors previously treated using SABR under breath hold were included in this IRB‐approved retrospective study as summarized in Table 1. Patients were evenly divided (*n* = 5) by number of lesions based on inclusion criteria for the SABR‐COMET Arm 1 (≤ 3 targets), SABR‐COMET Arm 2 (4−5 targets), SABR‐COMET‐10 (4−10 targets), and ARREST (> 10 targets) trials/study.

**TABLE 1:** Patient tumor characteristics. Statistics are displayed as (mean ± standard deviation [min, max]).

Trial	Case	Targets	Max Target‐to‐Target Distance (cm)	CTV Volume (cm^3^)	Distance to COM (cm)
**SABR‐COMET Arm 1**	1	2	14.9	1.05 ± 0.96 [0.37, 1.73]	7.5 ± 6.9 [2.6, 12.3]
	2	2	7.8	1.46 ± 1.14 [0.66, 2.27]	3.9 ± 3.0 [1.7, 6.0]
	3	2	3.8	12.19 ± 10.85 [4.51, 19.86]	1.9 ± 1.7 [0.7, 3.1]
	4	3	7.1	6.61 ± 9.84 [0.86, 17.97]	3.6 ± 2.7 [0.4, 5.3]
	5	3	26.3	4.27 ± 2.96 [0.93, 6.54]	12.6 ± 2.9 [10.9, 15.9]
**SABR‐COMET Arm 2**	6	4	17.6	0.94 ± 0.86 [0.36, 2.19]	9.9 ± 2.7 [6.1, 12.4]
	7	4	20.4	0.62 ± 0.62 [0.14, 1.53]	9.0 ± 4.7 [5.2, 15.4]
	8	5	15.3	4.93 ± 3.42 [1.00, 10.19]	6.2 ± 2.7 [3.3, 10.5]
	9	4	23.5	10.47 ± 18.52 [0.86, 38.24]	13.0 ± 8.4 [1.2, 21.0]
	10	5	19.0	1.69 ± 1.66 [0.61, 4.64]	7.3 ± 4.1 [2.9, 13.9]
**SABR‐COMET‐10**	11	6	21.7	2.14 ± 2.62 [0.31, 7.33]	10.8 ± 2.6 [7.9, 13.8]
	12	7	22.6	0.68 ± 0.20 [0.45, 1.00]	9.9 ± 2.6 [6.5, 12.6]
	13	8	22.4	1.26 ± 1.16 [0.24, 3.53]	9.8 ± 2.0 [7.3, 12.2]
	14	9	17.1	1.82 ± 1.90 [0.31, 5.81]	6.8 ± 1.7 [5.1, 10.3]
	15	9	24.6	0.22 ± 0.11 [0.07, 0.39]	9.9 ± 3.5 [3.8, 16.2]
**ARREST**	16	12	21.6	0.56 ± 0.32 [0.20, 1.18]	9.1 ± 1.7 [5.9, 11.5]
	17	13	32.4	1.42 ± 1.19 [0.19, 4.25]	12.8 ± 5.4 [4.9, 24.5]
	18	14	26.8	3.00 ± 3.78 [0.56, 13.44]	8.6 ± 3.7 [3.1, 16.8]
	19	14	13.2	4.46 ± 10.93 [0.19, 41.05]	4.8 ± 2.1 [2.4, 8.9]
	20	21	26.7	0.92 ± 0.60 [0.11, 1.88]	9.1 ± 4.0 [2.8, 19.0]

### 2.5 Evaluation versus uniform margin expansions

To evaluate whether uniform margins are sufficient for multi‐target treatments, the proposed k‐means based method was evaluated versus uniform margin expansions cited in prior studies. A 5‐mm margin as used in the SABR‐COMET trial,^4^ and a 10 mm margin used in a polymetastatic treatment planning study were evaluated.^15^ For each approach, the total margin volume was compared versus the k‐means method. The primary endpoint was the difference in total margin volume between methods, tested for statistical significance using a paired two‐sided Wilcoxon signed‐rank test. Comparisons were made versus the 5 and 10 mm margins, and the hybrid optimization method, with a Bonferroni correction applied and significance threshold of *p* = 0.0167 (0.05/3).

### 2.6 Loss of coverage due to rotational uncertainties

The additional margin required to maintain a set probability of target coverage for known rotational errors as a function of distance to isocenter may be computed according to Equation 6.

(6) $$\Delta m_{j}=\chi_{\alpha }\;\sqrt{{\sigma_{S}}^{2}+{\left(0.816d_{i,j}\sigma_{D}\frac{\pi }{180}\right)}^{2}}-\chi_{\alpha }\sigma_{S}$$

The extra margin (i.e., in addition to the 5 mm margin compensating for translational errors) required was computed for distances from isocenter up to 30.0 cm. The purpose of this was to advise on the selection of a single fixed margin to use for thoracic SABR, that would guarantee dose coverage for targets restricted within a maximum distance from isocenter, at the cost of additional normal tissue dose. The loss of CTV coverage that would occur with fixed margins of 5 mm, without additional margin expansion to account for rotational uncertainties, as a function of distance to isocenter, was also determined by sampling the chi‐square distribution function with three degrees of freedom at a value of, $\;{\left(\frac{\chi_{\alpha =0.05}\sigma_{S}}{\sqrt{{\sigma_{S}}^{2}+{\sigma_{j,R}}^{2}}}\right)}^{2}.$ ^7^

## 3 RESULTS

An automated framework for isocenter selection and margin determination was successfully implemented. Representative examples of isocenter placement and tumor clustering are shown in Figure S1, with corresponding distributions of individual target margins shown in Figure S2.

### 3.1 Total margin volume via k‐means

**FIGURE 3:**
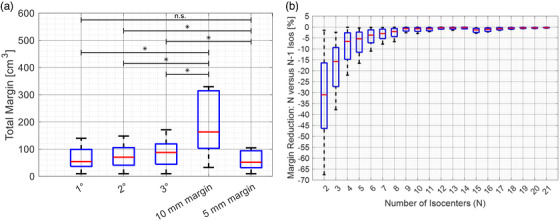
(a) The total target margin for rotational uncertainties ($\sigma_{D}$) of 1°, 2°, and 3° are shown versus fixed margins of 10 and 5 mm. Whiskers denote the 5th and 95th percentiles and the middle line denotes the median. Differences are denoted as significant (*) or nonsignificant (n.s.). (b) The percentage reduction in total margin volume for an additional isocenter, N, versus N‐1 isocenters is shown.

The distribution of total target margin volumes as a function of rotational uncertainties ($\sigma_{D}$ = 1°, 2°, 3°) are shown in Figure 3a versus fixed margins of 10 and 5 mm for all isocenter scenarios considered, with pairwise statistical comparison. The median total margin volume was 54.1 cm^3^, 70.3 cm^3^, and 87.6 cm^3^, for $\sigma_{D}$= 1°, 2° and 3°, respectively. For each scenario, the total target margin volume was significantly (*p*‐value < 0.0167) reduced relative to a fixed 10 mm margin which required a median total margin volume of 163.2 cm^3^. For $\sigma_{D}$ = 2° and $\sigma_{D}$ = 3°, the median total margin volume was significantly greater than the fixed 5 mm margin scenario (51.8 cm^3^). No significant difference in total margin volume was observed for $\sigma_{D}$ = 1° versus fixed 5 mm margins (*p*‐value = 0.067).

Figure 3b shows the percent reduction in the total margin volume that is attributed to adding an additional isocenter, aggregated across all patient cases. For example, use of two isocenters (*N* = 2) reduced the total margin volume by a median value of −31.0% (95th percentile = −67.6%, 5th percentile = −1.5%) compared to the single‐isocenter (*N* = 1) scenario. As the number of isocenters increased from *N* = 2 to *N* = 3, 4, 5, and 6, the median reduction in total margin decreased as −15.7%, −6.6%, −5.4%, and −3.7%, respectively. This demonstrates that diminishing returns are associated with increasing the total number of treatment isocenters, as progressively smaller reductions in total margin volume are achieved with each additional isocenter.

The reduction in total margin volume as a function of the number of isocenters are presented in supplemental Tables S1, S2, and S3 for each individual clinical case. The total margin volume for the single isocenter scenario is provided as well as the percent reduction in margin associated with each additional isocenter. The total number of determined isocenters is presented for thresholds of 10%, 20%, and 30%. Relative to the 10% threshold, the 20% and 30% thresholds required, on average, 1.2 and 1.8 fewer isocenters for 3.0° rotations, respectively, 0.9 and 1.3 fewer for 2.0°, and 0.7 and 1.0 fewer for 1.0°.

### 3.2 Validation of k‐means versus optimal isocenter total margin reduction

**FIGURE 4:**
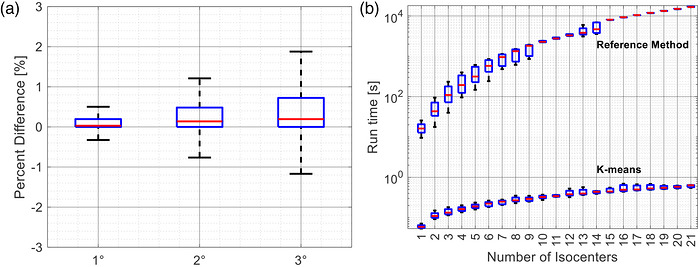
(a) The percentage difference of the k‐means versus the hybrid optimization method total treatment margin volume is summarized for all isocenter scenarios considered. Whiskers represent +/‐ 3σ capturing 99.7% of data. (b) Computational run times are plotted for k‐means and hybrid optimization methods versus number of treatment isocenters.

To evaluate the k‐means determined total margin volume versus the hybrid optimization technique, Figure 4a presents the percentage difference. The k‐means determined total margin volume was in close agreement with the hybrid optimization technique values with median differences of 0.03% ($\sigma_{D}$= 1°, *p*‐value = 0.940), 0.14% ($\sigma_{D}$= 2°, *p*‐value = 0.952), and 0.19% ($\sigma_{D}$= 3°, *p*‐value = 0.948). No significant differences were observed. All differences agreed within 1.2% at the 95th percentile and 1.9% at the 99.7th percentile (± 3 standard deviations). The median absolute differences in total margin volume were 0.01 cm^3^, 0.06 cm^3^, and 0.15 cm^3^ for rotational uncertainties of 1°, 2°, and 3°, respectively.

Figure 4b presents the computational run times for the k‐means and hybrid optimization methods as a function of the number of isocenters. Median run times were 0.19 s for k‐means and 306.9 s for the hybrid method, with run time increasing as the number of isocenters increased.

### 3.3 Loss of coverage due to rotational uncertainties

**FIGURE 5:**
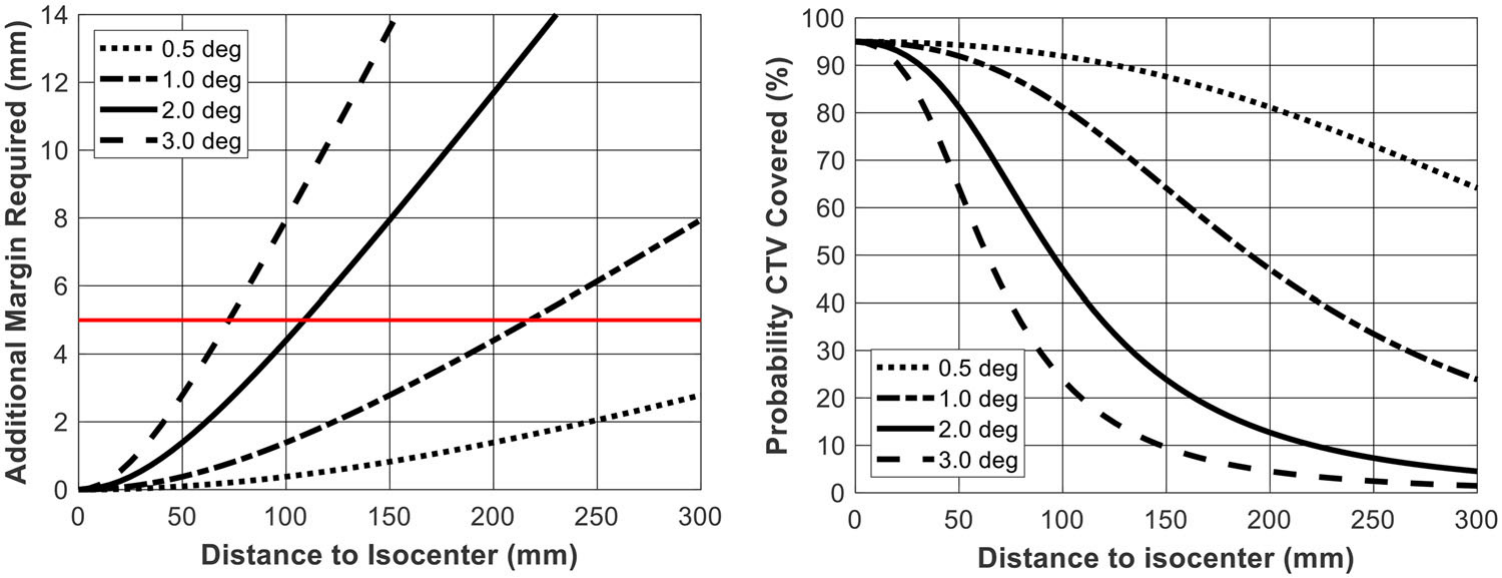
The extra margin required to maintain a set probability of CTV coverage is shown at left for assumed rotational uncertainties of 0.5°, 1.0°, 2.0°, and 3.0°. The probability of CTV coverage without additional margin is shown at right. An initial margin of 5 mm is assumed.

To provide clinical context at target‐to‐isocenter distances that may be encountered in multi‐target thoracic treatments, Figure 5 (left) presents the extra margin required to maintain a set probability of CTV coverage as the target distance from isocenter increases for varied rotational uncertainties. The red line shows the cutoff where coverage goals would not be met if an additional 5 mm margin was used to account for rotational uncertainties, corresponding to a 10 mm margin if 5 mm was sufficient for translational errors only. Figure 5 (right) demonstrates the loss of CTV coverage that would occur if additional margin expansion were not performed to account for the rotational uncertainties.

## 4 DISCUSSION

This work presents a computationally efficient method to simultaneously determine the number (N) of isocenter points, isocenter positions, target clusters, and treatment margins for multi‐target thoracic SABR. In the proposed framework, the k‐means clustering algorithm is applied to determine the locations of up to N isocenter positions to efficiently plan treatments for multiple thoracic tumor targets. The additional target specific treatment margins to account for rotational uncertainties are dynamically updated as a function of target distance to isocenter to maintain a set coverage probability.

The method was validated versus a hybrid optimization technique in terms of the total treatment margin volume in a cohort of twenty patients who previously received SABR for multiple thoracic tumors. No statistically significant differences in the total margin volume were observed. The median absolute difference in total margin volume did not exceed 0.15 cm^3^ which can be considered clinically negligible. Although it was previously demonstrated minimizing the squared Euclidean distance, as done with k‐means, does not guarantee a globally optimal solution,^13^ our results suggest that k‐means clustering provides near‐optimal and clinically acceptable solutions.

To implement in clinical practice, a dosimetrist, physicist, or physician could quickly evaluate the tradeoffs between total margin volume and the number of isocenters. Alternatively, a clinic could specify a threshold for the acceptable difference in absolute margin volume that is associated with using an additional isocenter, N, relative to N‐1 isocenters. If an additional isocenter results in a margin volume reduction that exceeds the threshold, it might be preferable to accept a longer treatment procedure with unique isocenters as a tradeoff of smaller margins. By automating the determination of isocenter positions and treatment margins, the k‐means based method could enhance both the efficiency and consistency of multi‐target treatment planning. Future work will focus on predicting or rapidly computing the dose distribution as a function of tumor dimensions, margins, and isocenters to directly evaluate dosimetric planning goals. Additionally, a retrospective analysis of volumetric treatment images, such as cone‐beam CTs, should be conducted to determine the magnitude of the rotational uncertainties assumed to vary between 1–3° in this study, and their relative contribution compared to other sources of uncertainty, such as intrafraction motion.^18^ Future work should also evaluate whether the assumption of normally distributed rotational errors is valid in the thoracic setting.

Two prior related studies have evaluated k‐means clustering for isocenter selection in multi‐target *intracranial* stereotactic radiosurgery (SRS) treatments: one limited to *single*‐isocenter treatments,^19^ while the other considered multi‐isocenter intracranial treatments with up to six targets and a maximum target separation of 135.5 mm.^14^ This work differs from those prior studies by considering up to 21 tumor targets, larger separation distances (i.e., up to 324.0 mm) associated with thoracic treatments, rotational uncertainties up to 3.0°, and introduces dynamically determined tumor margins as a function of distance to isocenter. In the extracranial setting, a recent guideline on delivering SABR for polymetastatic disease emphasized the practical challenges in isocenter selection and target grouping, while also acknowledging the considerations of margin selection and the potential for increased lymphopenia risk.^20^ Additionally, because total margin volume scales cubically with tumor radius, the developed method is particularly relevant for larger thoracic (Table 1) versus intracranial targets.

A limitation of this study is that performance was characterized exclusively in thoracic cancer patients. Although the method is geometrically general, further validation in other extracranial disease sites, such as the pelvis, abdomen, and spine, is warranted. A second limitation of this work is the exclusion of alternative clustering techniques such as k‐medoids, weighted k‐means, hierarchical clustering methods, or more advanced machine learning based approaches. Another limitation is that the hybrid optimization routine employed does not guarantee a global minimum. The optimization problem presented for multiple targets and large separation distances is inherently challenging, increasing both the search space and computation time. To address this, a stochastic simulated annealing optimization method with careful tuning of the temperature parameter, cooling scheme, and cold re‐starts was implemented to seek a global optimum. Future work could also explore brute force solutions on a large‐scale graphics processing unit computing cluster. Nonetheless, a clinically impactful finding from our study is that the k‐means framework generates solutions in under one second, while the hybrid optimization method varies significantly with the number of targets. Despite these limitations, this study demonstrates that k‐means clustering provides a computationally efficient and clinically effective solution for isocenter and margin selection in thoracic polymetastatic SABR treatment planning. Future work will focus on integrating this technique into a clinical treatment planning system to enable comprehensive dosimetric planning studies, including the evaluation of integral lung dose.

## 5 CONCLUSION

An end‐to‐end method was developed to cluster tumor targets and select isocenter locations for multi‐target thoracic SABR treatments while minimizing total margin volume. Validation against a hybrid optimization technique demonstrated that this method yields near‐optimal results, with clinically negligible differences in total margin volume.

## AUTHOR CONTRIBUTIONS

All authors made substantial contributions to this work and approved the final manuscript.

## CONFLICT OF INTEREST STATEMENT

The content is solely the responsibility of the authors and does not necessarily represent the official views of the National Institutes of Health. Funding acknowledged from the Badger Challenge and Wisconsin Alumni Research Foundation/Route 66 Ventures Cardiology Challenge. The other authors declare no conflicts of interest relevant to this work.

## Supporting information




Supporting Information

## Data Availability

The data that support the findings of this study are available from the corresponding author upon reasonable request.
