# Supplementary material for: Method for simultaneous selection of treatment isocenters and margins for polymetastatic extracranial stereotactic ablative radiotherapy
Source: J Appl Clin Med Phys. 2026 Jul 1;27(7):e70681. doi: 10.1002/acm2.70681 (PMC13322665; doi:10.1002/acm2.70681)
Supplement: Supplementary file 1 — Supporting Information [file ACM2-27-e70681-s001.docx]

**SUPPORTING INFORMATION**

**Figure S1:** The k-means clustering algorithm is demonstrated for three clinical cases. At left, all targets are treated with a single isocenter for patients with three (top row), five (middle), and fourteen (bottom row) targets. As the number of isocenters increases (left to right) the mean target distance to isocenter and associated uncertainties decrease, with a tradeoff of increased treatment time.


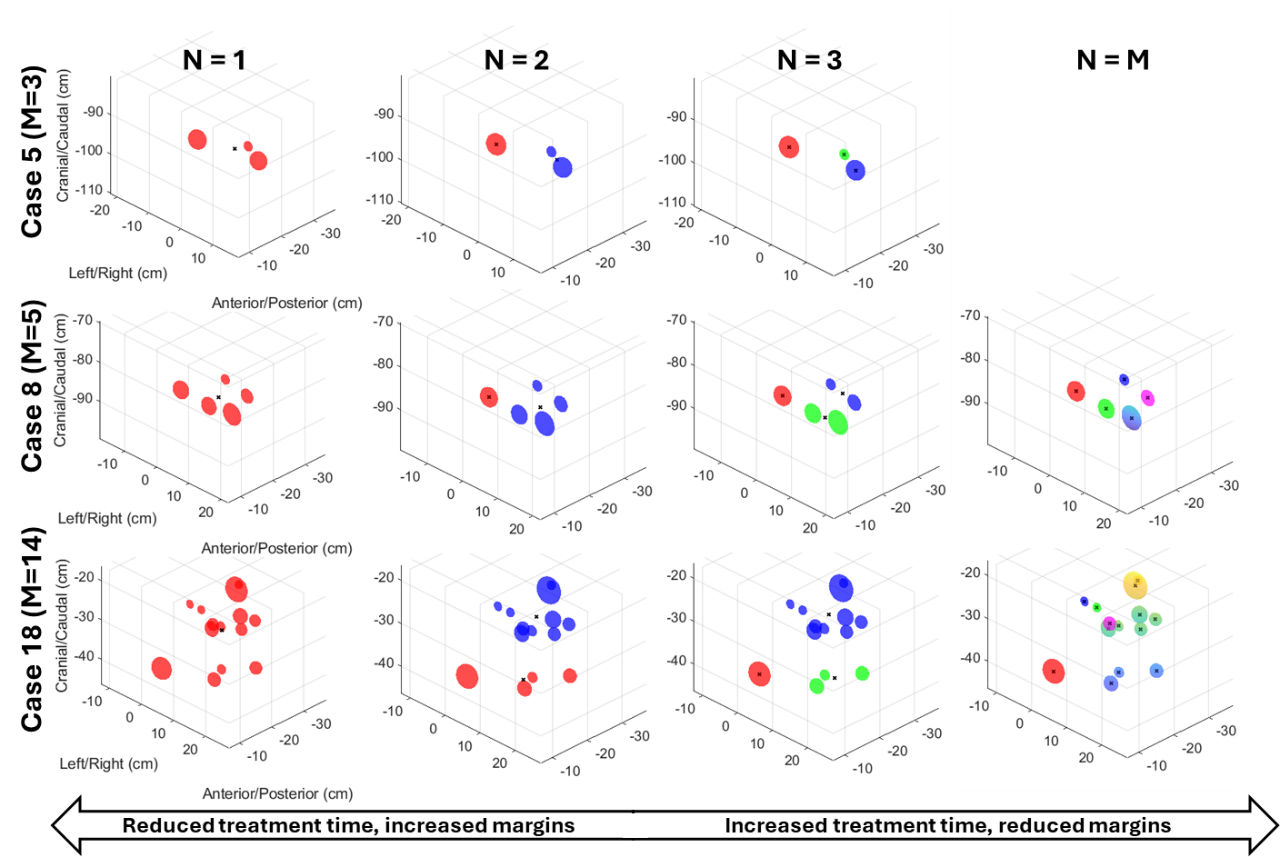


**Figure S2:** Individual target margins decrease with each additional planning isocenter. The isotropic margins are tumor specific and derived as a function of tumor distance to isocenter.


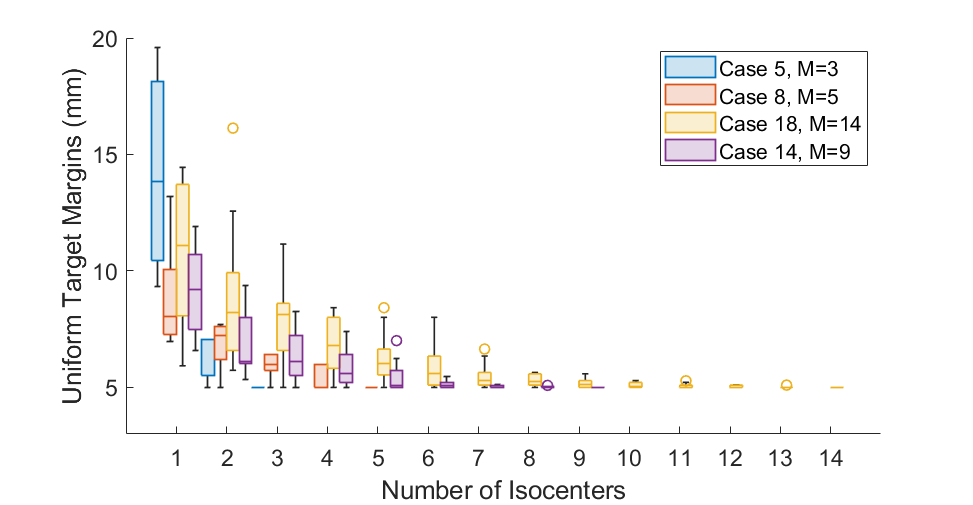

**Table S1:** The total margin volume versus the number of treatment isocenters with 1° rotational uncertainties for 20 clinical cases are presented. Margin reductions >10% due to an additional isocenter are highlighted in green, >20% in **bold**, and >30% in **italics**.

**Table S2**: The total margin volume versus the number of treatment isocenters with 2° rotational uncertainties for 20 clinical cases are presented. Margin reductions >10% due to an additional isocenter are highlighted in green, >20% in **bold**, and >30% in **italics**.

**Table S3:** The total margin volume versus the number of treatment isocenters with 3° rotational uncertainties for 20 clinical cases are presented. Margin reductions >10% due to an additional isocenter are highlighted in green, >20% in **bold**, and >30% in **italics**.
